# Detachment of the Retina

**Published:** 1892-01

**Authors:** Clarence C. Howard

**Affiliations:** New York, N. Y.


### DETACHMENT OF THE RETINA.

Read before the New York Homœopathic County Medical Society.

CLARENCE C. HOWARD, M. D., NEW YORK, N. Y.

MR. PRESIDENT, LADIES AND GENTLEMEN:

The following case came under my care April 20th, 1888. It presents so many features of interest because of its rarity of occurrence and the depleted condition of the patient, together with the fact that the condition had existed over two weeks under regular (?) treatment, I present it for your consideration. Mr. V., aged about fifty, given to habitual periods of intemperance for over thirty years; suffering from a chronic parenchymatous nephritis. Some years previously had a hemorrhage into the vitreous humor of the left eye, leaving a large patch of atrophy of the choroid and retina, and opacities of the vitreous which had in a measure interfered with the vision of that eye by producing a scotoma. While on a protracted debauch, having had several violent attacks of vomiting and retching, was suddenly seized with partial blindness of the right eye. The field of vision was restricted to a very small compass upward and outward; downward and inward, there was absolute loss of sight. Dr. Deady was called in consultation and diagnosed the detachment of the retina. An examination by the ophthalmoscope revealed the opaque gray folds of the retina quivering and trembling with every motion of the eye, the vessels were tortuous.

On this condition and symptoms, I prescribed Gelsem.$^{1M}$ one dose, and ordered the patient to bed, requiring him to lie flat on his back, with his head on a very small pillow, with particular instructions that he should make no muscular efforts whatsoever. Enemas of hot water were given to move the bowels, which were evacuated in a bed-pan, the urine being voided into a urinal. His diet consisted of milk and wheatena from the Health Food Company, the juice of beef and at intervals fruit, such as baked apples, oranges, etc. At the end of the first week the field of vision was very much extended and the ophthalmoscope showed a larger attached portion of the retina.

This improvement continued, and in six weeks the sight was normal. He was gradually allowed to resume the erect position, he first having been cautioned against any muscular exertion, and the use of the eye, which was positively forbidden. His sight remained perfect until the 3d of August, when without any apparent cause there was a recurrence of his trouble, but not to the extent of the first. He was again placed in the recumbent position and the same diet and precautions used as in the first instance, and one more dose of Gelsem.$^{1M}$ administered with, if anything, a more rapid recovery than in the first place.

On the 16th of August he complained of floating particles of black specks before the eye. Dr. Deady made another examination and found that a hemorrhage had taken place into the vitreous humor.

Kali-hyd.$^{200}$, one dose, was prescribed for this condition, with speedy relief.

The sight became normal and a careful examination by the ophthalmoscope revealed no lesions, the blood had been completely absorbed. The field of vision was perfect, the only remaining symptom was a peculiar condition of the sight, all objects appearing smaller. On this symptom and the mental condition Platina$^{45M}$ was given on September 14th, which brought about a perfectly natural and healthy condition of the eye.

It is a fact well worth mentioning that this worthy gentleman has been on no less than six drunken sprees in the interim without any recurrence of this trouble.

